# Patients’ perceptions of ethical issues in semi-autonomous robot-assisted surgery

**DOI:** 10.1007/s00464-026-13082-z

**Published:** 2026-07-24

**Authors:** Jie Ying Wu, Sydney Varnado, Ashley Leon, Camella J. Carlson, Alexander J. Langerman, Laurie L. Novak, Michelle L. Houston, Irene D. Feurer, Elisa J. Gordon

**Affiliations:** 1 Department of Computer Science, Vanderbilt University, Nashville, TN USA; 2 Department of Surgery, Vanderbilt University Medical Center, Nashville, TN USA; 3 School of Medicine, Vanderbilt University, Nashville, TN USA; 4 Department of Otolaryngology, Vanderbilt University Medical Center, Nashville, TN USA; 5 Department of Biomedical Informatics, Vanderbilt University Medical Center, Nashville, TN USA; 6 Department of Biostatistics, Vanderbilt University Medical Center, Nashville, TN USA; 7 Center for Biomedical Ethics and Society, Nashville, TN USA

**Keywords:** Qualitative interviews, Autonomy, Artificial intelligence, Decision-making, Informed consent, Information, Patient-centered care, Operation, Procedure

## Abstract

**Background:**

Artificial intelligence enables surgical robots to perform certain actions without surgeon control or semi-autonomously. Patient willingness to undergo surgery via an automated machine is a key barrier to adoption of emerging robotic technology. Further, semi-autonomous surgical robots raise numerous ethical concerns, yet little is known about patients’ perceptions of these issues. We assessed patients’ perceptions of ethical issues in semi-autonomous robot-assisted surgery (RAS) and how their perceptions would affect decision-making about undergoing semi-autonomous RAS.

**Methods:**

Adult patients who underwent surgery within the prior three years, with a non-autonomous robot or without any robot, were recruited via email. We conducted semi-structured teleconference interviews that included open- and closed-ended questions, to assess factors affecting patient decision-making and ethical concerns about semi-autonomous RAS, and demographics. Quantitative data were analyzed using descriptive statistics. Qualitative data were analyzed using thematic analysis.

**Results:**

Fifty patients participated. Most participants were female (62%), White (52%), and the mean age was 53. Most patients (56%) would undergo semi-autonomous RAS. However, willingness varied by which surgical process the robot would have control over. Many patients (46%) were highly willing to undergo semi-autonomous RAS when the robot has control over instruments. Four themes emerged about ethical concerns driving patients’ willingness to undergo semi-autonomous RAS: (1) desire for informed consent to express self-determination, (2) semi-autonomous RAS introduces new risks to procedures, (3) doctor-patient relationship and trust can facilitate patients’ willingness to undergo semi-autonomous RAS, and (4) semi-autonomous RAS comprises an important surgical innovation, conferring benefits.

**Conclusion:**

Our findings suggest that most patients would be willing to undergo semi-autonomous RAS. Willingness was related to ethical considerations of informed consent, perceived risks, level of surgeon versus robot control over surgical processes, doctor-patient relationship, and perceived benefits. Addressing patients’ perceptions of semi-autonomous RAS may facilitate greater integration of surgical robots into clinical practice.

**Supplementary Information:**

The online version contains supplementary material available at https://doi.org/10.1007/s00464-026-13082-z.

Robot-assisted surgery (RAS) has advanced the surgical field [1, 2] by providing better accuracy and precision [3–7]. Systematic reviews comparing RAS to laparoscopic and open partial nephrectomy, colorectal, and abdominopelvic surgeries found lower conversion to open surgery rate, blood loss, length of hospital stay, fewer complications, and more positive outcomes [3–6]. While most current commercially available surgical robots carry out the surgeon’s actions without exercising autonomy [2], the emergence of artificial intelligence (AI) has enabled surgical robots to make intraoperative surgical decisions [8].

Semi-autonomous robots can make critical decisions during surgery and potentially act without direct surgeon control [9]. Semi-autonomous robots have been used in *LASIK* eye surgery, hair transplant surgery, knee replacement surgery, and radiosurgery, typically carrying out surgical plans made by a surgeon. The nascent development of semi-autonomous surgical robots capable of more complex intraoperative decision-making raises new ethical considerations. Ethical concerns pertaining to informed consent, privacy, confidentiality, and changes in doctor-patient relationship remain largely unexamined and unresolved [10, 11].

Patients’ ethical concerns may influence their willingness to undergo semi-autonomous RAS. Little is known about patients’ perceptions of ethical issues. In a survey of postoperative patients (*n* = 383), patients were more likely than surgeons to consider all types of information about RAS essential for deciding whether to undergo RAS [12], with technical details about the procedure and the surgeon’s experience in performing the procedure driving patients’ informed consent. A survey of the general public (*n* = 727) found that most (72%) believed current RAS provided better outcomes, but half (55%) preferred conventional surgery [13], suggesting that factors beyond surgical outcomes shape treatment preferences [14]. While such surveys revealed the magnitude of patients’ decision-making factors, little is qualitatively known about what drives patients’ decision-making regarding undergoing semi-autonomous RAS. This study examined patients’ perceptions of ethical issues, hypothetical decision-making, and information needs for providing informed consent to undergo semi-autonomous RAS.

## Materials and methods

### Theoretical framework

The Health Information Technology Acceptance Model (HITAM) is a theoretical framework used to explain consumer acceptance and adoption of health information technology [15, 16]. HITAM posits that technology acceptance is driven by perceived ease of use, perceived usefulness, perceived enjoyment, attitude toward the technology, social influence, intention to use, system quality, and previous experience with technology. HITAM has been used to assess public perceptions of robotic technologies in surgery [17] and patients’ behavioral intentions toward robot-assisted gynecologic surgery [18]. We used the HITAM to inform our interview questions and analysis.

### Research design

We conducted a cross-sectional qualitative study of patient perceptions of ethical issues regarding semi-autonomous RAS. We conducted semi-structured interviews with surgical patients. We used the Standards for Reporting Qualitative Research for quality reporting of qualitative methods and data. [19]

### Setting

The study was conducted at Vanderbilt University (VU) and Vanderbilt University Medical Center (VUMC); data were collected at VUMC from April 2025 to July 2025. The Institutional Review Board at Vanderbilt University Medical Center approved the study and served as the single IRB (IRB #241967).

### Study population and recruitment

Eligible individuals included adult (≥ 18 years of age), English-speaking patients who had undergone urology/renal, thoracic, gynecology, oncology, head and neck, or orthopedic procedures at VUMC within 3 years prior to recruitment, with or without robot assistance, and had an email address. We oversampled for under-represented minorities and for women to ensure representation given racial and sex differences in perceptions of RAS [20, 21]. Potential participants were recruited via email with up to five follow-up phone calls to ascertain interest and schedule an interview.

### Data collection

We conducted semi-structured interviews by teleconference (Microsoft Teams) to assess patients’ perceptions of ethical concerns regarding semi-autonomous RAS, using standardized approaches (i.e., interview guide). At the beginning of each interview, the interviewer presented a brief introduction to current, non-semi-autonomous surgical robots, and to semi-autonomous surgical robots in development. Interviews included 32 open- and 7 closed-ended questions on topics including: perceptions of semi-autonomous surgical robots, decision-making, informed consent, doctor-patient relationship, privacy and confidentiality, and concluded with demographic and health-related questions. Closed-ended questions were recorded through the Research Electronic Data Capture (REDCap) [22]. We conducted an initial set of cognitive interviews (*n* = 10) to refine question wording and enhance the clarity of the interview guide [23]. We avoided the term AI throughout the interview guide to focus on the physical embodiment and immediate impact of the surgical robot that leverages AI to perform surgical processes, rather than focus on other AI applications in healthcare (i.e., diagnostics, transcription), which have been previously examined at length.

Closed-ended questions included two sets of 5-point Likert scale questions. One set assessed patients’ willingness to undergo semi-autonomous RAS across six types of surgical processes: (1) planning of surgery, (2) surgical decision-making, (3) instruments, (4) considering patient preferences, (5) changes in plans like halting the procedure, and (6) decisions that could create disability. Responses were anchored by “not at all willing,” “a little bit willing,” “somewhat willing,” “quite a bit willing,” and “extremely willing.” Two additional 5-point Likert scale questions separately assessed the level of control that patients would prefer surgeons and the level of control that patients would prefer the robot to maintain over the six aforementioned surgical processes, anchored by “no control,” “equal control,” and “total control.”

We administered the attitude and behavioral intention questions developed as part of the HITAM, which includes six 5-point Likert scale questions about attitudes and behavioral intentions toward health information technology, which has been shown to have excellent internal consistency reliability (Cronbach’s alpha > 0.90) [16]. For each participant, we summed the items within each domain (attitude and behavioral intention) and then calculated overall average scores for each domain. The range of the HITAM items is 3–15. Interviews were audio-recorded and typically lasted 45–60 min. Participants were compensated with a $50 electronic gift card.

### Qualitative data analysis

All audio-recorded interviews were transcribed using Teams AI, which were reviewed by staff while listening to the audio-recorded interviews. Corrected transcripts were then analyzed for themes that emerged from the data [24] using the constant comparison [25] and inductive and deductive coding methods based on the interview questions [26]. Transcripts were iteratively and independently reviewed by members of the team until reaching thematic saturation to establish the codebook. The research team used an iterative process to independently review and openly code the first set of 3 transcriptions. After coding each set, the research team held analytic forums to compare codes and resolve discrepancies to reach consensus in code definitions for the codebook [27]. Four transcripts were independently coded to establish inter-rater reliability (Kappa > 0.80) between three study team members [28]. Once inter-rater reliability was reached, two research team members independently coded each transcript to increase the rigor and reproducibility of the qualitative findings. Thereafter, two research team members prepared code summaries based on the analysis of all coded segments for a given code to generate themes. While a sample of *n* = 15 is sufficient for grounded theory studies to reach theoretical saturation [29], we sought, a priori, to recruit *n* = 50 patients to enable broader analysis of shared meanings, and analysis by demographics, consistent with ethnographic research [30, 31].

### Statistical and mixed-methods analysis

Descriptive statistics were used to summarize responses across the respondent sample. Likert scale responses to willingness questions were dichotomized as: low willingness (“not at all willing,” “a little bit willing,” and “somewhat willing”) versus high willingness (“quite a bit willing” and “extremely willing”). Likert scale responses to level of control questions were dichotomized as low control (“no control” to “equal control”) and high control (greater than “equal control” and “total control”). We used binomial regression to assess associations between categorical and continuous variables. Chi-squared tests or Fisher’s Exact Tests were used to assess associations between dichotomous variables (i.e., willingness to undergo semi-autonomous RAS) and demographic and clinical characteristics [32]. All tests were non-directional, and *p* < 0.05 was considered statistically significant. Jamovi software, version 2.6 (The Jamovi project, Sydney, Australia) was used to perform statistical analyses.

## Results

**Fig. 1:**
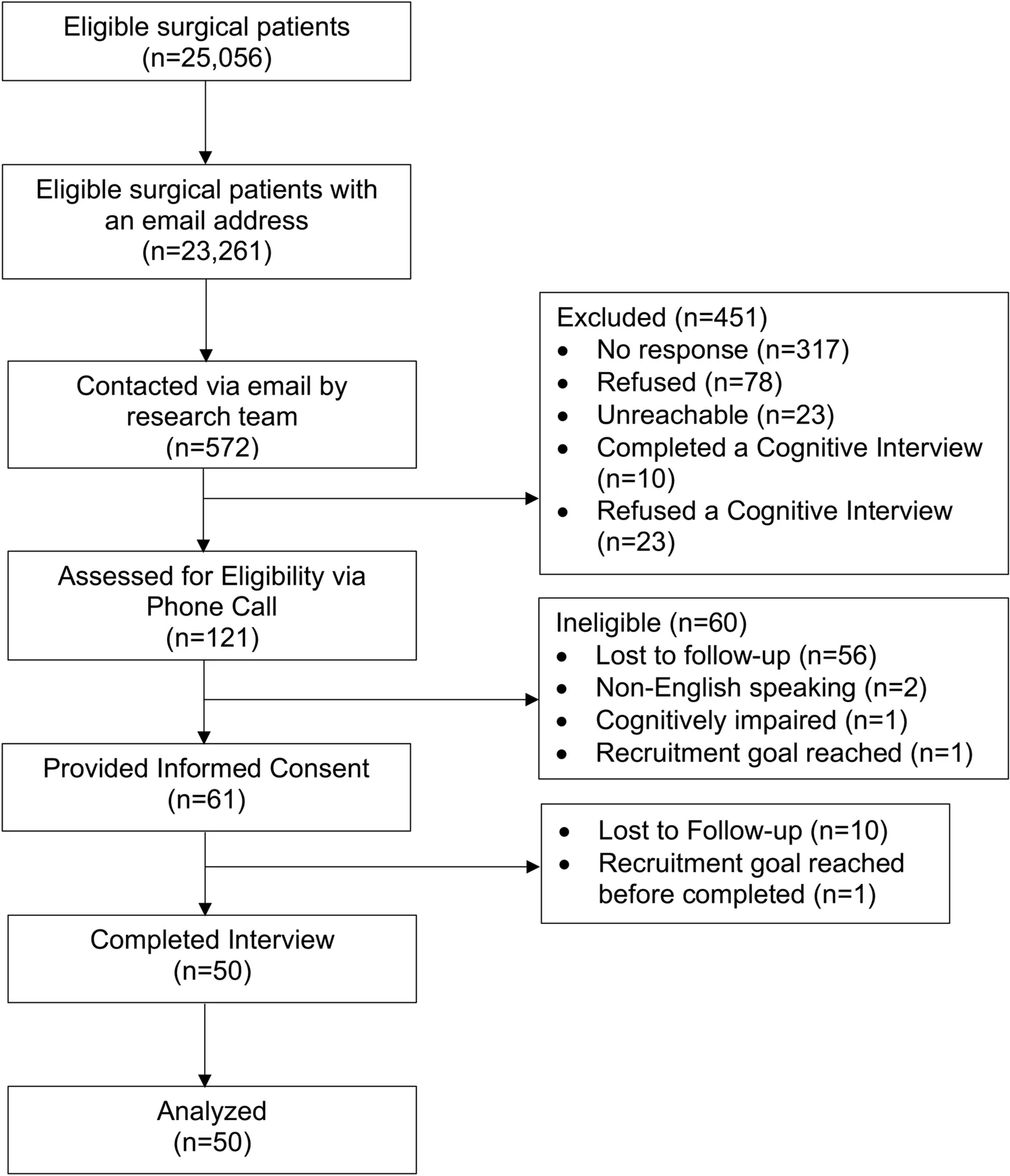
Consort diagram

Of all eligible surgical patients (*n* = 25,056), *n* = 23,261 (92.8%) had an email address (Fig. 1). Of these, we reached out to *n* = 572 patients, of whom *n* = 451 were excluded, resulting in *n* = 121 who were assessed for eligibility. Of these, *n* = 60 were ineligible and *n* = 61 provided informed consent. Of these, a total of 50 patients participated in an interview (9.7% participation rate). Most were female (62%), White (52%) or Black (42%), and had a median age of 53 years (Table 1). The three leading types of surgery patients underwent included: urology (24%), general (16%), and gynecology (10%) procedures. Participants (*n* = 46) had their last surgery a mean of 411 days (SD = 372, range: 11–1826) before their interview. Most patients (84%) had heard about surgical robots before the interview.

**Table 1:** Participant characteristics, *N* = 50

Characteristic	Total *n* (%)
Median age, years [IQR]	53 [40, 60]
Sex	
Female	31 (62)
Male	19 (38)
Highest education level	
High school graduate, GED, or equivalent	7 (14)
Some college	10 (20)
College graduate	17 (34)
Post-graduate degree (MA, PhD, MD, DO, etc.)	16 (32)
Marital status	
Married/domestic partner/civil union	27 (54)
Never married/single	18 (36)
Separated or divorced	4 (8)
Living with partner	1 (2)
Race	
White	26 (52)
Black or African American	21 (42)
Asian	2 (4)
Middle Eastern or North African	1 (2)
Other	1 (2)
Employment status	
Employed full-time	24 (48)
Retired	14 (28)
Not employed	4 (8)
Disabled	3 (6)
Employed part-time	2 (4)
Student	1 (2)
Homemaker	1 (2)
Prefer not to answer	1 (2)
Annual income	
Less than $15,000	3 (6)
Between $15,000 and $34,999	4 (8)
Between $35,000 and $54,999	7 (14)
Between $55,000 and $74,999	4 (8)
Between $75,000 and $94,999	6 (12)
More than $95,000	20 (40)
Prefer not to disclose	6 (12)
Primary health insurance coverage	
Private health insurance	35 (70)
Medicaid/medicare	13 (26)
Other	2 (4)
Health literacy	
Adequate	43 (86)
Inadequate	7 (14)
Health status	
Excellent	3 (6)
Very good	15 (30)
Good	19 (38)
Fair	12 (24)
Prefer not to answer	1 (2)
Number of patients who underwent each type of surgery	
Urology	12 (24)
General	8 (16)
Gynecology	5 (10)
Orthopedic	4 (8)
Bariatric	3 (6)
Colorectal	3 (6)
Endocrine	3 (6)
Hepatobiliary	3 (6)
Spine	3 (6)
Thoracic	2 (4)
Head and neck	1 (2)
Oncology	1 (2)
Ophthalmology	1 (2)
Otolaryngology	1 (2)
Underwent robot-assisted surgery	
Yes	23 (46)
No	19 (38)
I don’t know	8 (16)
Technology comfort level	
I am among the first of my peers to adopt the latest technology	8 (167)
I like to see how new technology works for others before I adopt it	22 (46)
I tend to wait until the technology is well established before I adopt it	17 (35)
I reluctantly adopt new technology	1 (2)
Computer comfort level	
Not at all comfortable	1 (2)
Somewhat comfortable	7 (15)
Moderately comfortable	15 (31)
Completely comfortable	25 (52)
Computer games comfort level	
Not at all comfortable	13 (27)
A little comfortable	2 (4)
Somewhat comfortable	12 (25)
Moderately comfortable	9 (19)
Completely comfortable	11 (23)
Prefer not to answer	1 (2)
Rapid change in health technology comfort level	
Not at all comfortable	2 (4)
A little comfortable	5 (10)
Somewhat comfortable	15 (31)
Moderately comfortable	10 (21)
Completely comfortable	16 (33)
Chat GPT knowledge	
A lot	21 (45)
A little	16 (34)
Nothing at all	10 (21)
Use of ChatGPT to learn something new	
Yes	22 (63)
No	13 (37)
Use of ChatGPT for entertainment	
Yes	17 (49)
No	17 (49)
No answer	1 (3)
Use of ChatGPT for tasks at work	
Yes	14 (40)
No	21 (60)
HITAM mean (standard deviation)	
Overall attitudes	12.4 (2.8)
I am positive about using HIT to manage my health and to search for reliable health information	4.1 (1.1)
I think it is beneficial to manage my health and search for reliable health information using HIT	4.3 (0.9)
I am satisfied by and large with the use of HIT to manage my health and search for reliable health information using HIT	4.1 (1.0)
Overall behavioral intentions	12.3 (2.8)
I will continue to use HIT to manage my health and to search for reliable health information	4.2 (1.0)
I will regularly use HIT to manage my health and to search for reliable health information	4.1 (1.1)
I will recommend use of HIT to other people to manage their health and to search for reliable health information	4.0 (1.0)

### Factors that would influence patients’ decisions to undergo semi-autonomous RAS

**Fig. 2:**
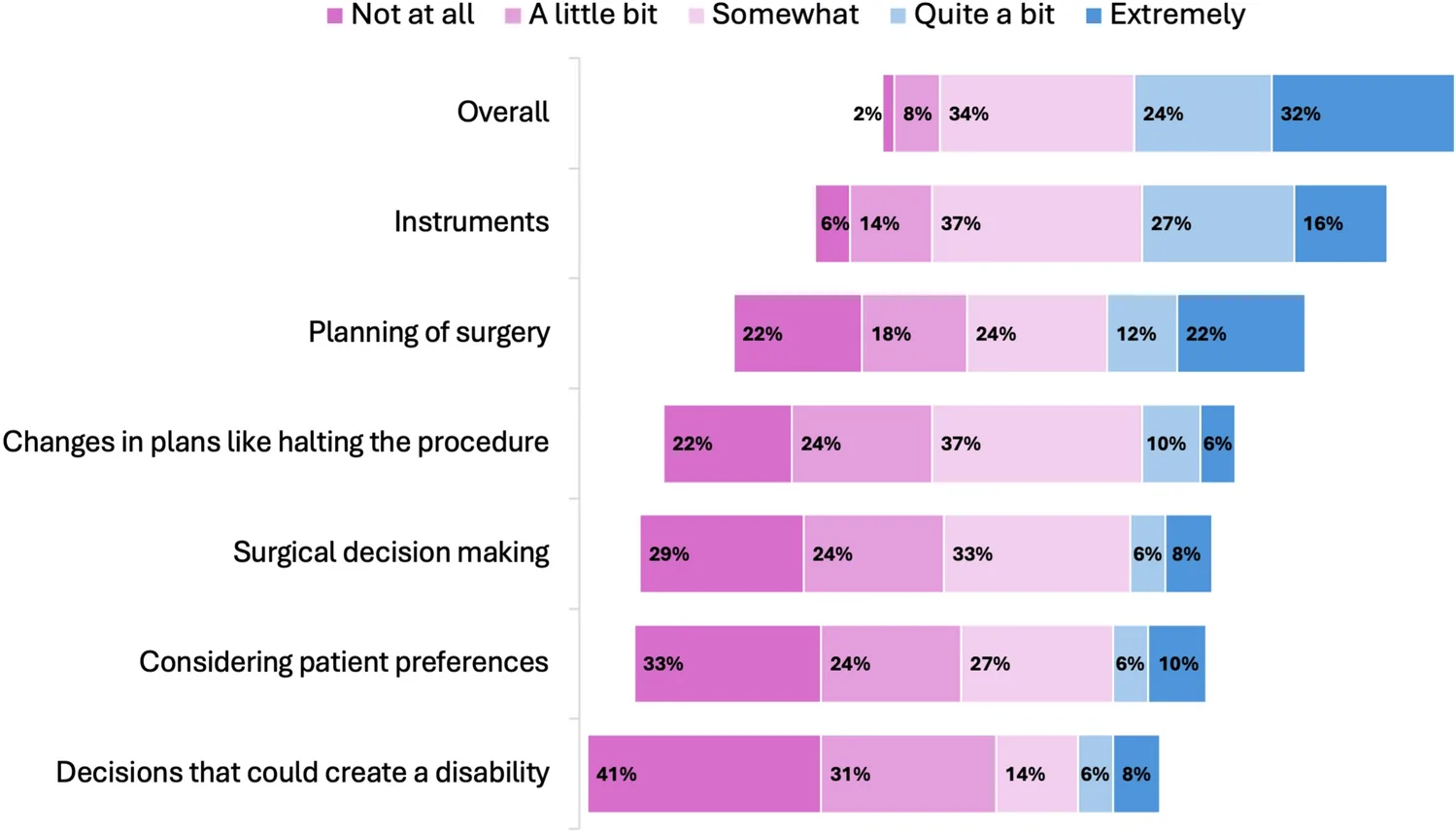
Percent of participants by their willingness to undergo semi-autonomous robot-assisted surgery and by the type of control the robot would have over the different types of surgical processes

More than half of participants (56%) were ‘quite a bit’ or ‘extremely’ willing to undergo semi-autonomous RAS (Fig. 2). Participants’ willingness to undergo semi-autonomous RAS varied by the type of control the semi-autonomous robot would have over different types of surgical processes. For example, more participants would be ‘quite a bit’ or ‘extremely’ willing to undergo semi-autonomous RAS when the robot had control over instruments (43%) than when the robot had control over decisions that could create a disability (14%). No demographic or clinical variable was found to be significantly related to overall willingness to undergo semi-autonomous RAS (all *p* > 0.05).

**Fig. 3:**
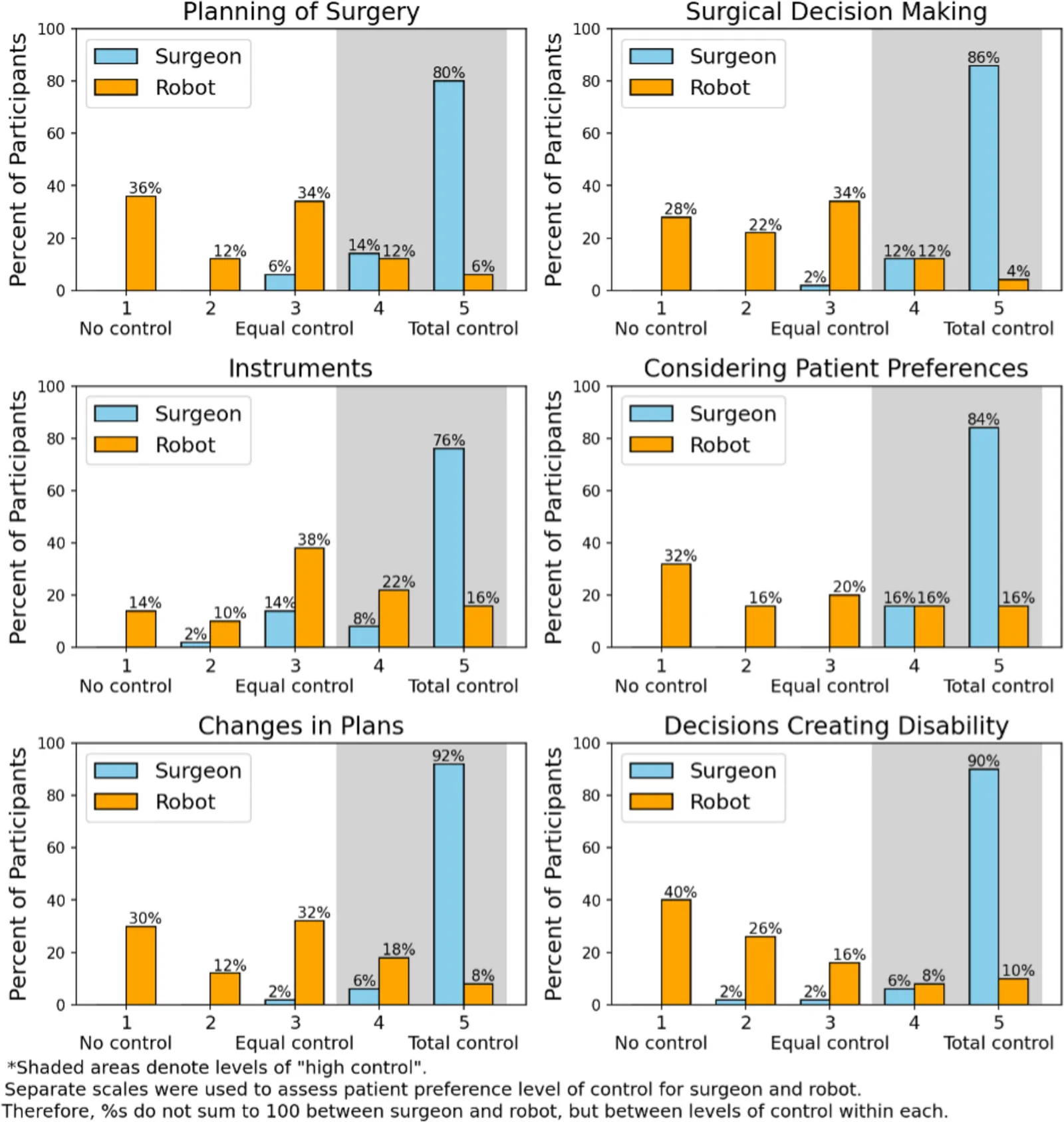
Participant preference for how much control the surgeon and robot should exert over different surgical processes*

In questions separately asking patients to rate how much control over different types of surgical processes they would prefer either surgeons or robots to have, nearly all patients consistently desired surgeons to have a high level of control across all surgical processes. Approximately a third of participants supported the robot having high levels of control in two surgical processes: instruments (38%) and considering patient preferences (32%) (Fig. 3). Patients’ rationale for rating control over various surgical processes centered on the “human” elements a surgeon provides, such as empathy and personal connection, and the surgeon’s expertise from extensive training and hands-on experience. Patients expressed a lack of trust in robots to replicate these qualities. However, some patients acknowledged that robots could serve as valuable tools when used with a surgeon, particularly for surgical planning, changing plans, and controlling instruments (Supplemental Table 1).

Patients reported overall positive attitudes and behavioral intentions toward health information technology in the HITAM Likert Scale questionnaire. Forty-seven patients completed the HITAM questionnaire and had an average of 12.4 (SD = 2.8) for attitude and an average of 12.3 (SD = 2.8) for behavioral intention categories.

In open-ended questions, participants reported benefits of semi-autonomous RAS as the main reason for their willingness to undergo semi-autonomous RAS (Table 2). Patients reported less willingness to undergo semi-autonomous RAS for more complex procedures due to the lack of confidence in the robot’s abilities. Participants stated their overall willingness to undergo semi-autonomous surgery is higher for surgery with a perceived lower risk of complications (e.g., appendectomy) compared to surgery with a higher risk of complications (e.g., brain surgery).

**Table 2:** Representative Illustrative quotations

Themes/subthemes	Quotations
Overall willingness to undergo semi-autonomous RAS	“Well, I think it would depend on what kind of surgery it was—how—like complicated or… if you’re taking out somebody’s appendix, you know, that should be pretty easy to have an autonomous robot assist with. But, if you’re doing, I don’t know, triple bypass, I’d be a little shaky on letting a robot just do that all by itself, you know?. The depth and risk involved, like if it’s brain surgery, if it’s, you know, something big that if it’s messed up, well, you’re just screwed for life. Then I would be less likely to submit to that than if it was taken out tonsils or taken in an appendix or, you know, something that is really common and not as likely to impair you for life.” 59-year-old female (ID#MC91060*)*
Patients desired a thorough informed consent process to express self-determination Importance of expressing self-determination for semi-autonomous RAS	“You should know what’s going on inside your body. Additionally, you should be made aware that an artificial intelligence is being used in there and it’s not completely up to the surgeon’s control. Some people are looking for a doctor. They want that particular surgeon because of his choices, and if a choice is being taken out of a human’s hands, someone should. They’ll be aware that that is going to be taking place inside of them, especially if it’s a life or death situation.” 38-year-old male (ID#MC91063)
Ensuring that surgical robots do no harm (nonmaleficence) Harm from robot malfunction would deter patients of semi-autonomous RAS	“I’m concerned that something might go wrong with … the computers that guide the robot. You know, some sort of glitch that might make it too late for the surgeon to correct. And so, the risk [is] of the robot doing something that is unexpected. … And of course, you know, that the possibility of injury or death that might come from that.” 53-year-old female (ID#MC91021)
Patients desired reassurance of surgeon oversight of surgical robots Participants expected surgeon involvement in their surgery even when the robot carries out the surgical process	“You know, the surgeon or the doctor’s skill set should still be there. And the expertise should still be there and you don’t want to have surgeons or medical providers that are relying more or more so on the robot’s diagnosis or assumptions instead of their own.” 40-year-old female (ID#MC91022)
Participants expected surgeon involvement in their surgery even when the robot carries out the surgical process	“I’ll ride in an autonomous car, but I don’t necessarily want a fully autonomous robot operating on me while I’m under anesthesia, but semi-autonomous as long as there’s a human. The human interaction is making the final decision.” 40-year-old female (ID#MC91068)
Participants expected surgeon involvement in their surgery even when the robot carries out the surgical process	“I would not be as comfortable if it was a robot doing the whole surgery as I would if there was a person connected to it.” 53-year-old female (ID#MC91021)
Surgeons’ over-reliance on robots could harm patients	“That kind of scares me. I feel like with any kind of technology. …. I feel like that takes away from the doctor’s education, and we might start relying on the semi-autonomous robots a little too heavily. And then, you know what? If something went wrong and does the surgeon actually know how to finish the surgery? You know, if they’ve relied on the robot to do it all up until that point, I don’t know. I have a negative feeling about the semi-autonomous one… We would become too reliant on it. I would prefer the human surgeon to have the expertise to have the knowledge to work on another human body and not rely on the robot.” 46-year-old female (ID#MC91030)
Lack of personal connection with robots may curb willingness to undergo of semi-autonomous RAS	"You can’t have a personal connection with a machine. Where my surgeon, Doctor [surgeon’s name], was warm and made me feel confident before the surgery. You know, I felt like I trusted her like we had built a relationship after visiting and all of that and her associates, there was one name [associate]. I mean, all the associates that worked for her that were in the surgical room made me feel comfortable and safe and a robot cannot do that.” 53-year-old female (ID#MC91021)
Perceived benefits of semi-autonomous RAS enhanced surgical performance	“I was scheduled for about the 5th or maybe the 6th surgery of the day. And I didn’t want that. I didn’t want a physician to be operating on four or five people before me. … I just need the full attention. A semi-autonomous robot kind of takes [on] some of that wearing down of the day. I don’t want to be 8 h into a surgery with a tired surgeon, so I don’t think I’d have any hesitation.” 53-year-old male (ID#MC91017)
Enhanced surgical performance	“If they have a procedure plan and they’re comparing it to, you know, millions of surgeries or 10 s of thousands of surgeries before and they can calculate it based on, you know, your age, your weight, your all the other conditions and all other variables compiled in there and kind of adjust and give feedback for that for a physician to review going in there is huge. There’s only so much information that the human brain can hold and access. And so having a semi-autonomous robot that’s being able to pull data is–it would be a big win for me.” 53-year-old male (ID#MC91017)
Reduced surgeon fatigue	“When I had my brain surgery, the technology would have been nice back then to take some of the pressure off the doctor, the surgeon, because I was in surgery for such a long time over the amount that possibly having the robot there to pinpoint a little more would alleviate some of the long stress. My surgery was almost 16 h. That’s a long time for a surgeon to do that, and I know specifically, even though he had attendings there, he was the only one who did it. So having the robot there would definitely take some of the pressure off the surgery situation” 64-year-old female (ID#MC91025)
Reduced human error/bias	“I know there’s some machine error too, but from a human error standpoint, I never know what’s going on in the physician’s personal life outside of. I know they’re dedicated. I know they’re there. But, if there’s always a human error component, that always makes me a little more nervous than anything else. And a semi-autonomous robot, I don’t think it’s going to get tired. It’s not going to have wear down.” 53-year-old male (ID#MC91017)
Improved accessibility of surgery	“Smaller areas, smaller hospitals like mine where I live would have access to exceptional surgical care because of, you know, eventually those could be purchased by remote locations, remote hospitals. I traveled 4 h to go to [hospital]. If that were an option, you could get it done in your own town. It would be very handy to have that.” 53-year-old female (ID#MC91021)

Participants referred to their positive prior experience with robotic surgery as justification for their willingness to undergo semi-autonomous RAS. Conversely, patients cited negative prior experience with robotic surgery as a reason for their reservations about undergoing semi-autonomous RAS. Participants varied in whether their friends and family would support them in undergoing semi-autonomous RAS, and in whether participants would consider their friends and family’s opinions in their decision. Friends’ and family’s opinions would not influence some participants’ decisions because friends and family lacked medical expertise. By contrast, friends’ and family’s opinions would strongly influence other patients because their families have made medical decisions that had good outcomes in the past. Overall, patients emphasized that their decision would depend on their own perception of what was best for their health.

### Ethical issues

Participants reported a range of ethical issues, from none to extensive, with semi-autonomous RAS, which informed their willingness to undergo semi-autonomous RAS. Relatively few participants did not perceive ethical concerns with semi-autonomous surgical robots and characterized the robots as neutral tools, “just another tool in the toolbox.” However, other patients identified several ethical issues regarding how the robot may change surgical care. Four themes emerged regarding patients’ ethical concerns about undergoing semi-automated RAS, including: (1) patients desire informed consent to express self-determination, (2) semi-autonomous RAS introduces new risks to procedures, (3) doctor-patient relationship and trust can facilitate patients’ willingness to undergo semi-autonomous RAS, and (4) semi-autonomous RAS comprises an important surgical innovation, which confers many benefits. Representative illustrative quotations are presented in Table 2.

## Theme 1: Patients desired a thorough informed consent process to express self-determination

Participants consistently emphasized the importance of providing informed consent for undergoing semi-autonomous RAS. As one patient stated, “[patients] should know as much as they can about the surgery and who and what is performing it,” and some reckoned that not all patients want to have robots involved in their surgery. Subthemes that emerged included the importance of self-determination, patients’ information needs, and the role of the robot versus the surgeon.

### Subtheme A: Importance of expressing self-determination for semi-autonomous RAS

Participants stated that patients should provide input on whether a semi-autonomous robot is used during the procedure, grounded in a patient’s right to decide what happens to their body. Participants emphasized that patients should have bodily autonomy, or “control over what someone is doing on their body” and how it is performed, since the surgeon will not be performing all of the surgical processes. Participants commonly maintained that patients should be able to “decide for themselves” about robotic assistance and believed that it is a “personal decision.” Participants imagined other patients refusing semi-autonomous RAS given ethical concerns, religious beliefs, personal assessments of risks and benefits, and hesitancy about new technology, but did not expound on the rationale for these points.

### Subtheme B: Patients desired information about semi-autonomous RAS

In a closed-ended question, the three leading types of information patients reported needing the most to make an informed decision about undergoing semi-autonomous RAS included: how the robot will be used in the surgery (98%), the risk of unexpected complications (88%), and the surgical team’s experience performing RAS (86%) (Table 3).

**Table 3:** Types of information patients reported needing to make an informed decision about undergoing semi-autonomous RAS, *N* = 50^1^

Information topics	Yes^2^ *N* (%)
1. How the robot will be used in the surgery	49 (98)
2. The risk of unexpected complications resulting	44 (88)
3. The surgical team’s experience performing robot-assisted surgery	43 (86)
4. The potential risks of using semi-autonomous surgical robots	41 (82)
5. The category of control that the robot will have during surgical procedure	39 (78)
6. Evidence that semi-autonomous surgical robots work	39 (78)
7. Whether data about the patient are collected and used in training the robot	38 (76)
8. The level of autonomy the robot will have during the surgical procedure	37 (74)
9. How the surgeon will fix robot system failure	37 (74)
10. The methods used to minimize robot harms to patients	37 (74)
11. Alternative treatments to using surgical robots	37 (74)
12. How data collected about the patient are kept confidential	36 (72)
13. Number of times this semi-autonomous robot has performed the surgery (to account for learning)	35 (70)
14. Information about the company that makes the robot	30 (60)
15. The limitations of the robot’s capacities	30 (60)
16. The number of patient cases needed for surgical teams to gain competency in using surgical robots	28 (56)
17. Use of anonymous data for tracking safety of this surgical robot	22 (44)
18. Payment to the company that made the robot by the surgeon or institution using the robot	11 (22)

^1^1 participant chose not answer Q4, Q5, Q6, Q8, Q12, Q15, and Q18

^2 ^This column reflects participants reporting that they would desire being informed about this information topic

In open-ended questions, participants expressed the need for a broad range of information to make informed treatment decisions about undergoing semi-autonomous RAS. Participants considered a thorough informed consent process that covered these information needs, and the option to opt out of undergoing semi-autonomous RAS would support patients’ self-determination.

Patients specifically desired information about surgeons’ prior experience, the robot’s role during surgery, and safety and maintenance protocols. Participants reported that their surgeon’s experience performing the surgery, both with and without semi-autonomous RAS, should be disclosed during the informed consent process, given its relevance to patients’ decision-making. Participants also desired knowing how long the particular robot that would be operating on them had been in use since its adoption into clinical practice (i.e., number of years in service) and the frequency of use. Participants considered these statistics important because they did not want to be among the first people on whom the robot had been used, either in general or by their surgeon. Participants also highlighted the need for statistical information regarding the surgical robot’s performance. Desired statistical information included the robotic system’s success and failure rates, technological malfunctions, and complication rates following the surgical procedure. Patients regarded all technology as fallible and desired to know the contingency plan in case of malfunctions.

Patients also emphasized their need for information about the semi-autonomous surgical robot’s safety protocols and regular maintenance. Participants expected that adequate robot testing and maintenance would provide assurance of safe surgical robot use. Participants recommended that the robot’s testing standards should be made available to patients as part of the informed consent process. Participants said that this information could help them feel more prepared and less anxious heading into surgery.

### Subtheme C: Informed consent disclosure should cover the use of a surgical robot

Participants commonly reinforced their right to know “who or what is operating” and to receive a “clear understanding about the instrument.” Patients emphasized their need to understand how the robot works, how it will be used, and the robot’s capabilities. Participants compared the need for surgeons to disclose the use of surgical robots during surgery to their disclosing a trainee’s involvement in surgery, acknowledging the patient’s desire to know the different operators, whether robotic or human, and the operators’ experience and capabilities. Participants also expressed interest in knowing what a surgeon anticipates happening during surgery and that not all steps are completed solely by the surgeon if a semi-autonomous robot is used for assistance.

## Theme 2: Ensuring that surgical robots do no harm (nonmaleficence)

Patients anticipated potential malfunctions in surgical robots based on their previous experience with technology. Concerns that patients identified included: (1) surgical robots’ malfunctions may harm patients due to the small margin for error in surgery, and (2) operating teams need to be properly trained to use the new technology. Patients identified thorough testing protocols for the robot and training protocols for operating teams as ways to alleviate these concerns.

### Subtheme A: Harm from robot malfunction would deter patients from semi-autonomous RAS

Patients commonly reported concerns about the safety of semi-autonomous RAS. Patients reported fears about the risk of the robot malfunctioning, which could potentially cause surgical error, patient injury, or death. Patients expressed apprehension that many technologies they use daily malfunction and that surgical robots may also be prone to malfunctions. Participants noted how robots could “short circuit,” “glitch,” experience an “electrical issue,” suffer a power or Internet outage, or be vulnerable to a security risk like hacking, which could contribute to machine failure. They desired details about how robots underwent testing for approved use on patients. Patient acceptance of semi-autonomous RAS relied on the demonstration of technological reliability in clinical settings, where the margin for error is small.

### Subtheme B: Surgical training is an important component of safety

Participants commonly urged caution about training surgeons to use surgical robots. Participants questioned whether semi-autonomous surgical robots would be safe under a surgeon’s control or whether human error, including “inputting wrong information” or pressing the wrong button, could harm the patient during surgery. Participants perceived training for semi-autonomous RAS as critical to ensure safe surgical robot procedures. Participants desired knowing that the surgical team had followed thorough training protocols for using semi-autonomous surgical robots to feel assured about undergoing semi-autonomous RAS.

## Theme 3: Patients desired reassurance of surgeon oversight of surgical robots

Participants highlighted the importance of surgeons’ oversight of semi-autonomous surgical robots during surgery. Participants conveyed this point in 4 ways: (1) surgeon involvement, (2) surgeon’s overreliance on the robot, (3) trust in surgeons, and (4) lack of personal connection with the robot.

### Subtheme A: Participants expected surgeon involvement in their surgery even when the robot carries out the surgical process

Participants expected that their surgeon would remain actively involved throughout the procedure to ensure safe use of the robot, adapt to intraoperative changes, oversee surgical decisions, and intervene if necessary. Participants desired knowing who was performing each surgical process: the surgeon or the robot. All participants expected the surgeon to be physically present during the procedure, even if the robot performed surgical actions, given that participants placed value on the surgeon’s medical knowledge. Participants desired the surgeon’s oversight and expertise throughout the procedure because they perceived that semi-autonomous RAS could increase the procedure’s risk.

### Subtheme B: Surgeons’ overreliance on robots could harm patients

Participants feared that surgeons might become dependent on surgical robots for decision-making and technical skills and lose their surgical acumen. Specifically, patients anticipated that surgeons’ over-reliance on robots might erode surgeons’ skills to the point of the surgeon becoming “obsolete.”

Consequently, such dependence would render surgeons unable to perform the surgery without the robot, which may harm future patients if surgeons need to take over the procedure upon robot malfunction. Additionally, patients questioned whether surgeons would become de-skilled with the adoption of semi-autonomous surgical robots. If surgeons become reliant on robots to perform large portions of a procedure, patients questioned whether surgeons could take over the procedure in the event of a malfunction.

### Subtheme C: Patients’ trust in surgeons would affect decisions about undergoing semi-autonomous RAS

Patients emphasized their trust in their surgeons and their surgeons’ ability to make the best choices for the patient’s care. Participants related how trust in their surgeon’s recommendation would be critical for their decision-making about whether to undergo semi-autonomous RAS. Participants reported that they would therefore be more inclined to undergo surgery if their surgeon thoroughly explained the process of RAS to them or recommended it.

### Subtheme D: Lack of personal connection with robots may curb willingness to undergo semi-autonomous RAS

Patients expressed concerns about the inability to form a personal connection with the robot to establish trust and build a relationship. Given the importance of the doctor-patient relationship in treatment decision-making, the lack of a patient–robot relationship may curb patients’ willingness to undergo semi-autonomous RAS. Since patients viewed robots as unable to form personal connections and express emotions, patients worried that robots could not provide the same level of care as their surgeon.

## Theme 4: Perceived benefits of semi-autonomous RAS

During the interview introduction, the research staff presented several benefits of semi-autonomous RAS, some of which particularly resonated with patients. For example, patients reported enhanced surgical performance, improved precision, accuracy, speed, and efficiency of the procedure as notable key benefits of semi-autonomous RAS. Participants also said they liked how semi-autonomous RAS could generate better surgical outcomes through quicker recovery and minimal bleeding.

### Subtheme A: Enhanced surgical performance

Participants attributed the possibility of better outcomes to robots by enabling less invasive surgery given the use of fewer and smaller incisions. Participants appreciated the robots’ ability to navigate challenging anatomic spaces inaccessible to surgeons and provide surgeons with better visualization. A few patients believed that by moving in a steadier manner, the robot’s incisions would be more accurate, resulting in faster surgical procedures.

### Subtheme B: Reduced surgeon fatigue

Participants commonly noted that semi-autonomous RAS would decrease surgeons’ fatigue by reducing the physical strain of operating. Participants considered how the surgical robot could reduce the surgeon’s workload, ease the physical demand on the surgeon, and reduce medical errors. According to participants, reducing surgeon fatigue could benefit the patient by making procedures more consistent and reducing negative outcomes.

### Subtheme C: Reduced human error/bias

Participants cautioned that human error or bias could occur if the surgeon performed the procedure, but use of a surgical robot could mitigate this potential. Respondents commonly perceived semi-autonomous robots as more “objective,” resulting in less human error, and subsequently, posing less harm to patients during surgery than surgeons.

### Subtheme D: Improved accessibility of surgery

Some participants acknowledged that if surgeons were unavailable, robots could increase patients’ access to surgical procedures. While all participants stated a preference for the surgeon to be in the room during surgery, patients also acknowledged how robots could benefit patients in locations with little access to surgical care. Robots may provide more options for patients who cannot or would prefer not to travel to receive specialized surgical care.

## Discussion

In this mixed-methods study, we identified several factors affecting patients’ willingness to undergo semi-autonomous RAS, including patients’ ethical concerns. A key finding was that more than half of participants were willing to undergo semi-autonomous RAS. Willingness depended on the surgeon’s versus the robot’s level and type of control during the procedure. It was noteworthy that participants reported greater willingness to undergo semi-autonomous RAS when the robot maintained high levels of control over surgical instruments, while patients reported less willingness when robots maintained more control over surgical decision-making. Applying a patient-centered perspective to engineering surgical robots suggests that the design and development of surgical robots should be informed by patient willingness based on different types of control. Increasing surgical robot capabilities in types of control that patients are more willing to accept first could increase patient adoption of semi-autonomous RAS.

We found that patients expressed the need for different types of information in order to provide informed treatment decisions about undergoing semi-autonomous RAS, similar to prior research on robotic surgery [12, 33]. Disclosing how the robot is tested and how the surgical team is trained is essential to increase patient comfort with new technology [12]. The shift from a fully surgeon-operated surgical robot to semi-autonomous surgical robots requires a renovation of the informed consent process given the additional considerations of procedures (i.e., the role of the surgeon versus the role of the robot), risks (i.e., malfunction), and benefits (i.e., enhanced surgical performance) that must be disclosed. A survey study of 440 potential total knee arthroplasty patients’ opinions of robot-assisted total knee arthroscopy found similar perceptions of benefits and risks [33]. Most frequently mentioned benefits included more accurate surgery, better outcomes, and faster recovery. Most frequently identified concerns included harm from malfunction, reduced surgeon role in the procedure, and lack of supportive research. Our findings highlight the need for patient education materials that facilitate patient adoption of semi-autonomous RAS. While patients recognized that surgical robots could improve surgical outcomes, most patients did not perceive robots as being able to replace surgeons, citing a belief that robots cannot build trust.

Understanding patient preferences of levels of control by the semi-autonomous robot during semi-autonomous RAS may enhance patients’ informed treatment decision-making process. Having more information would help patients express their autonomy and self-determination over what happens to their body.

Patients’ willingness to undergo semi-autonomous RAS may be informed by hospital policies and regulatory frameworks regarding semi-autonomous RAS. Research found that patient acceptance of high-risk novel medical procedures depended on the institutional environment in which the procedure was performed and the regulatory requirements. [34]

Patients expressed concern about surgeons becoming deskilled by over-reliance on semi-autonomous RAS. This concern is justified given evidence from other areas of medicine. For example, AI use reduced endoscopists’ ability to detect adenoma in current non-AI-assisted colonoscopy [35]. Hospitals with surgical robot programs should consider the implications of relying heavily on semi-autonomous RAS on surgeons to become deskilled and patient willingness to undergo RAS. [34]

Based on qualitative responses, family and friends would influence some participants’ willingness to undergo semi-autonomous RAS. Quantitative responses reinforce these findings. Attitudes toward supporting technology are based on other factors including system reliability, social influence, perceived ease of use, perceived threat, and perceived usefulness [16]. We also found system reliability, perceived threat, and perceived usefulness to be important factors in participants’ willingness to undergo semi-autonomous RAS. Other studies corroborate our findings. An online survey found that patients’ preference for semi-autonomous RAS over fully autonomous RAS was primarily driven by concerns regarding system reliability and perceived threat [17]. A different cross-sectional survey found perceived ease of use (defined by patients’ perception of the ease of understanding robotic surgery) to be the primary driving factor, followed by perceived usefulness, as factors affecting behavioral intentions toward robot-assisted gynecologic surgery [18]. Neither previous survey found social influence to be a significant factor toward attitude and behavioral intention toward robotic surgery.

A benefit of qualitative research is the identification of assumptions that people take for granted. In this study, we found that a handful of patients (mis)perceived that the use of surgical robots would result in faster surgery, which they perceived as beneficial because of the reduction in anesthesia introduced by prolonging operating time.

Other quantitative research found that patient preference for RAS was associated with patient acceptance of RAS [33]. Future research should examine how surgeons can optimally address patients’ concerns about the semi-autonomous surgical robot’s capabilities, benefits, and risks in ways that foster the procedure’s and the robot’s trustworthiness and enhance patients’ informed consent.

### Strengths

Our sample patient population was large for a qualitative study, enabling greater depth and breadth of analyses. Our sample patient population included a large representation of racially/ethnically minoritized populations.

### Limitations

As a single-center study, our findings may not be transferable to patients at other institutions. However, our inclusion of patients who had undergone different types of surgical procedures provides insights into a broad array of surgical experiences.

Replicating the study at multiple sites may clarify transferability of study findings. Although most eligible surgical patient participants had an email address (92.8%), selection bias may have occurred, favoring those who had access to digital technology.

## Conclusion

Our findings suggest that most patients would be willing to undergo semi-autonomous RAS. Many factors affected surgical patients’ perceived willingness to undergo semi-autonomous RAS, relating to ethical considerations of informed consent, perceived risks, level of surgeon versus robot control over surgical processes, doctor–patient relationship, and perceived benefits. Addressing patients’ ethical concerns and disclosing desired information may optimize the informed consent process. Addressing patients’ perceptions of semi-autonomous RAS may facilitate the integration of surgical robots into clinical practice.

## Supplementary Information

Below is the link to the electronic supplementary material.

Supplementary file1 (DOCX 23 KB)

## Data Availability

The datasets generated during and/or analyzed during the current study are available upon eligibility determination in the Qualitative Data Repository [https://doi.org/10.5064/F6DN5P6I].
