# Supplementary material for: Patients’ perceptions of ethical issues in semi-autonomous robot-assisted surgery
Source: Surg Endosc. 2026 Jul 24;40(9):8026–41. doi: 10.1007/s00464-026-13082-z (PMC13582349; doi:10.1007/s00464-026-13082-z)
Supplement: Supplementary file 1 [file 464_2026_13082_MOESM1_ESM.docx]

| **Supplemental Table 1. Patients’ Considerations of Surgeon versus Robot Control over Surgery by Categories of Control** | | | | | | | | |
| --- | --- | --- | --- | --- | --- | --- | --- | --- |
| Control Category | Humanistic Factor | General Preference for Surgeon | Surgeon Expertise | Shared Control | Lack of Trust in Robot’s Ability | Robot as a Tool | Patient-Doctor Relationship and Trust | Surgeon Responsibility |
| Disability | XXX |  |  |  |  |  | X | XX |
| Considering Patient Preferences | XXX | X |  |  | X |  | XX |  |
| Changes to Surgical Plan | XXX | XX | XX |  |  | X |  |  |
| Surgical Planning | XX | XXX | XX | X |  | X |  |  |
| Decision-Making | X | XX | XX | X | X |  |  |  |
| Instruments |  | XXX | X | XX | X | X |  |  |
| *Frequency of the theme appearance was coded as ‘XXX’ if n ≥ 15, ‘XX’ if 10 ≤ n < 15, and ‘X’ if 5 ≤ n < 10. | | | | | | | | |
